# Experiments and Observations Made on Animals with the Hydrogenous Sulphurated Gas

**Published:** 1802-12-01

**Authors:** 


### *Experiments and Observations made on Animals with the Hydrogenous Sulphurated Gas*; *by* Cit. CHAUSSIER.

THE mineral waters into the compofition of which fulphur enters, fuch as the waters of Bareges, of Cauterets, &c. are frequently employed by phyficians with confiderable fuccefs.

Thofe who eftimate the power of remedies entirely by the nature of the fubftances which enter their compofition, and which can be extracted by analyfis, have attributed the advantages derived from thefe waters, entirely to the fmall quantity of hydrogenous fulphurated gas which they contain. This opinion, fupported by fome chemical facts, has added much to the fuppofed medical virtues of this gas, and has led to the inference, that in a pure or gafeous ftate its virtues muft be much greater than when diluted largely in water; and it has been imagined, that introduced into the ftomach, or applied to the furface, the fame combinations fhould be formed as in the elaboratory of the chemift, without any experiment whatever to prove its action on animal life, or without reflecting how much the effects of a fubftance are changed by the dofe in which it is adminiftered. The interior ufe of hydrogenous fulphurated gas has been recommended in affections of the ftomach, and of the lungs; its efficacy as an antidote to arfenic has been highly fpoken of, and its external ufe in difeafes of the fkin, &c. has been ftrongly recommended.

Berthollet has warned us againft the frequent abufe of chemical reafoning on Medicine; analogies drawn from the curfory branches of Medical Science but too frequently lead us from the effential object of a Phyfician, namely, the contemplation of the vital powers, the energies, and the laws of animal life.

Thefe confiderations have led Cit. Chauffier to determine by experiment, the effects of the hydrogenous fulphurated gas, as well pure as diluted in water, on living animals. Although it had been already afcertained, that an animal died immediately on infpiring hydrogenous fulphurated gas, Citizen Chauffier thought it neceffary to repeat the experiments, the better to obferve the phenomena.

Different animals were fubmitted to the action of this gas, every one of whom expired after fome feconds,* and every means

---

* Frogs were found to be an exception, as they continued to live fome minutes. This Cit. Chauffier accounts for; Firft, by the firm denfe texture of the fkin, covered with a mucous, preventing the action of the gas into which they were plunged. Secondly, by the peculiar difpofition of the mouth, noftrils, means of resuscitation proved ineffectual. On examining animals killed by this gas, it was observed, 1st. That the cavities of the nose and bronchiæ were covered by a brownish mucus. Secondly, That the blood had undergone a remarkable change, being black and thick, an effect extending to every part which received many blood-vessels; thus the lungs, liver, spleen, and even the encephalon were found changed. Thirdly, The muscles were not only changed in colour, but this contractile power was always diminished, and in some cases totally lost. Fourthly, All the soft parts had lost their consistence, quickly putrefied, and emitted an uncommonly bad smell;* and as a proof of the extensive influence of this gas when taken into the lungs by respiration, a plate of silver, or a piece of white oxyd of lead, which had been introduced under the skin, became black when the animal had inspired. The baneful effects of the hydrog. sulp. gas taken into the lungs being proved, Cit. Chaussier thought it necessary to ascertain its effects on other organs of the body, and even when applied to different surfaces. Among a number of experiments made with this view, the following are most remarkable.

Exp. I. A bladder filled with hydrog. sulph. gas was attached to a small tube with a valve; the extremity of the tube was insinuated through a small opening under the skin in the thigh of a rabbit; a portion of the gas was pressed into the subcutaneous cellular substance, so as to form an artificial emphysema; as soon as the gas had ascended as high as the thorax (i. e. in about 30 seconds) the animal gave a shriek and dropped dead; a variety of stimuli were immediately applied, but the animal showed no signs of remaining life. The same experiment on frogs was followed by the same results. All the superficial cellular substance, and even the superficial muscles were changed to a dark brown.

In a second experiment it was found that water saturated with this nostrils, glottis, and lungs, moderating and changing the order of respiration, and which may be remarked when the animal is immersed in any noxious fluid. He contracts his mouth and nostrils, throws into the cavity of the throat, which is very dilatable, the air which had been in the lungs and submitted to their action, and which he frequently draws in again to the lungs, by this means using every particle necessary to life. It is not until after some minutes that the animal is under a necessity of renewing the air; so that in fact the frog does not live longer in this air than other animals, if, before he is plunged into it, his trachea be opened, or his glottis destroyed.

* Mr. Hunter had already remarked the same want of consistence in the parts of animals that had been killed suddenly, as by lightning, &c. and we have observed it eminently in the muscles, &c. of people who had suffered capital punishment.

this gas produced the fame violent effects; and to prove that thefe confequences were peculiar to the hydrog. fulph. gas, different other fluids, fuch as atmofpheric air, water, hydrogen gas, oxygen, carbonic acid gas, &c. were introduced with the fame apparatus, and except fome trifling mechanical inconvenience from the operation, the fluid was abforbed, and no remarkable confequence enfued. The hydrog. fulph. gas thrown into the rectum of a rabbit, killed it in a few feconds; the inteftines, liver, and adjoining vifcera were changed in colour, but the contents of the thorax and head were not altered. This experiment was repeated on a horfe, in the prefence of a number of perfons; the animal firft endeavoured to expel the gas, to prevent which care was taken; he was feized with convulfion, and died in the fpace of a minute. The immediate opening of the animal fhewed the fame changes of colour as in the preceding experiments. Water impregnated with this air, was injected into the anus of different animals, and in different dofes of the gas, all of whom died within the fpace of two minutes. Cit. Chauffier determined to afcertain the effects of this gas on the ftomach, which he did by laying bare the œfophagus on the fide of the neck, and introducing with the apparatus already defcribed, the gas pure, and water impregnated with it; the operation was almoft inftantaneoufly followed by death. The colour of the inner membrane of the ftomach was much changed, the other vifcera were not affected.

*Paris, Nov.* 12, 1802.

[ To be continued. ]

